# Ovarian Dropsy

**Published:** 1846-04

**Authors:** 


*Ovarian Dropsy.*—Mr. Eager, in the ***Provincial Medical and Surgieal Journal***, narrates the case of a young married woman labouring under ovarian dropsy, in whom the cyst ruptured, and extravasated its contents into the peritoneum, inducing subacute peritonitis. The inflammation was subdued by appropriate treatment, and the absorption of the effused fluid went on rapidly. The patient recovered.—*Ibid.*

